# Prussian blue nanozyme-mediated nanoscavenger ameliorates acute pancreatitis via inhibiting TLRs/NF-κB signaling pathway

**DOI:** 10.7150/thno.52010

**Published:** 2021-01-01

**Authors:** Xue Xie, Jiulong Zhao, Wei Gao, Jie Chen, Bing Hu, Xiaojun Cai, Yuanyi Zheng

**Affiliations:** 1 Department of Ultrasound in Medicine, Shanghai Jiao Tong University Affiliated Sixth People's Hospital, Shanghai, 200233, P. R. China; 2 Department of Gastroenterology, Changhai Hospital, Second Military Medical University, Shanghai 200433, P. R. China.; 3 Shanghai Institute of Ultrasound in Medicine, Shanghai Jiao Tong University Affiliated Sixth People's Hospital, Shanghai, 200233, P. R. China.; 4 Chongqing Key Laboratory of Ultrasound Molecular Imaging, Ultrasound Department of the Second Affiliated Hospital of Chongqing Medical University. Chongqing 400010, P. R. China.

**Keywords:** prussian blue nanozyme, acute pancreatitis, inflammation, reactive oxygen species, oxidative stress

## Abstract

**Rationale:** Acute pancreatitis (AP) is a serious acute condition affecting the abdomen and shows high morbidity and mortality rates. Its global incidence has increased in recent years. Inflammation and oxidative stress are potential therapeutic targets for AP. This study was conducted to investigate the intrinsic anti-oxidative and anti-inflammatory effects of Prussian blue nanozyme (PBzyme) on AP, along with its underlying mechanism.

**Methods:** Prussian blue nanozymes were prepared by polyvinylpyrrolidone modification method. The effect of PBzyme on inhibiting inflammation and scavenging reactive oxygen species was verified at the cellular level. The efficacy and mechanism of PBzyme for prophylactically treating AP were evaluated using the following methods: serum testing *in vivo*, histological scoring following hematoxylin and eosin staining, terminal deoxynucleotidyl transferase dUTP nick end labeling fluorescence staining, polymerase chain reaction array, Kyoto Encyclopedia of Genes and Genomes analysis and Western blotting analysis.

**Results:** The synthetic PBzyme showed potent anti-oxidative and anti-inflammatory effects in reducing oxidative stress and alleviating inflammation both* in vitro* and *in vivo* in the prophylactic treatment of AP. The prophylactic therapeutic efficacy of PBzyme on AP may involve inhibition of the toll-like receptor/nuclear factor-κB signaling pathway and reactive oxygen species scavenging.

**Conclusion:** The single-component, gram-level mass production, stable intrinsic biological activity, biosafety, and good therapeutic efficacy suggest the potential of PBzyme in the preventive treatment of AP. This study provides a foundation for the clinical application of PBzyme.

## Introduction

Acute pancreatitis (AP) is one of the most common acute conditions affecting the abdomen 1. The global incidence of AP is currently increasing. Additionally, serious disease occurs in 20-30% of patients with AP and the disease has a mortality rate of 40% 2, 3. In AP, pancreatic enzymes are activated and cause digestion, edema, hemorrhage, and even necrosis of the pancreatic tissue 3-6. Currently, AP is managed mainly by supportive and targeted prevention and treatment of systemic complications. Although AP has been widely examined, the clinical outcome of patients with AP has not greatly improved over the years. No effective treatment or clinically approved drug is available for treating AP. Therefore, safe and effective strategies for the intervention and treatment of AP are urgently needed. Activation of nuclear factor (NF)-κB is considered as a key inflammatory pathway in the pathogenesis of AP 7. In AP, NF-κB leads to cell injury and local inflammation 7. The inflammatory cascade is initiated in acinar cells by the activation of NF-κB before the onset of the innate immune response. The unbalanced redox state not only causes oxidative damage, but also upregulates the expression of interleukin (IL)-1β, IL-6, and tumor necrosis factor (TNF)-α as intracellular signals by activating NF-κB. Inflammatory cytokines (IL-1, IL-6, and TNF-α) play a central role in the progression of AP 6-9 and are mainly produced by activated macrophages, lymphocytes, and fibroblasts. Additionally, oxidative stress plays a major role in AP, causing a large number of macrophages to infiltrate into the inflammatory area. Oxidative stress is mainly caused by NADPH oxidase and mitochondrial dysfunction during AP. Hydrogen peroxide (H_2_O_2_) is the second messenger of NADPH oxidase and main source of reactive oxygen species (ROS) in inflammation 10-13. Oxidative stress of neutrophils activated during the inflammatory response to acinar injury may cause the further spread of local and systemic inflammation 5, 14. Thus, NF-κB activation and oxidative stress are potential therapeutic targets for AP 5, 15.

Currently, the drugs targeting NF-κB and oxidative stress for the treatment of AP include curcumin 14, 16, visnagin 17, shikonin 18, and lycopene 19, which are plant extracts that can reduce the level of oxidative stress and inhibit the activation of NF-κB. These plant extracts show good pharmacological effects and greatly alleviate the symptoms of AP. However, the preparation and treatment processes of these drugs are complex, and their clinical applications remain limited by the lipid solubility of the drug and other pharmacokinetic/pharmacodynamics challenges 5, 15. Alternatively, nanomaterials with enzymatic catalytic properties (nanozyme) have been developed in recent years 20, 21. Nanozymes have various advantages as antioxidants such as their easy modification, low cost, and high stability, and thus have been being widely applied in biomedical field 20. Nanozymes can drive antioxidant enzyme-like activities to scavenge intracellular overexpressed ROS and alleviate inflammation 22. Nanoceria can switch between the oxidized state of Ce^3+^ and Ce^4+^, and thus may block oxidation and inflammation signals involved in the pathogenesis of AP through its catalase (CAT)- and superoxide dismutase (SOD)-like activities 23. However, the CAT and SOD properties of ceria nanoparticles are affected by many factors, such as the size, Ce^3+^/Ce^4+^ ratio and surface area of nanoceria 24. Additionally, the biosafety of these nanozymes is considered a hindrance in the treatment of AP 25, 26. Prussian blue (PB) has been approved by the FDA to treat poisoning with thallium and other radioactive elements in the clinic. Using the controllable properties and structure of PB, we previously constructed hollow mesoporous PB nanoparticles to efficiently diagnose and treat tumors 27-29. The bioactivity of PB for scavenging ROS 29 was consistent with that in other reports 30, 31. In inflammatory bowel disease and ischemic stroke models, Prussian blue nanozyme (PBzyme) drove intrinsic ROS scavenging and inflammation inhibiting properties to achieve good protective and therapeutic efficacy 32, 33. This preliminary therapeutic exploration lays the ground work for the study of PBzyme in AP and provides an idea for further exploring the mechanism, considering that inflammation and oxidative stress are two important therapeutic targets of AP.

**Scheme 1:**
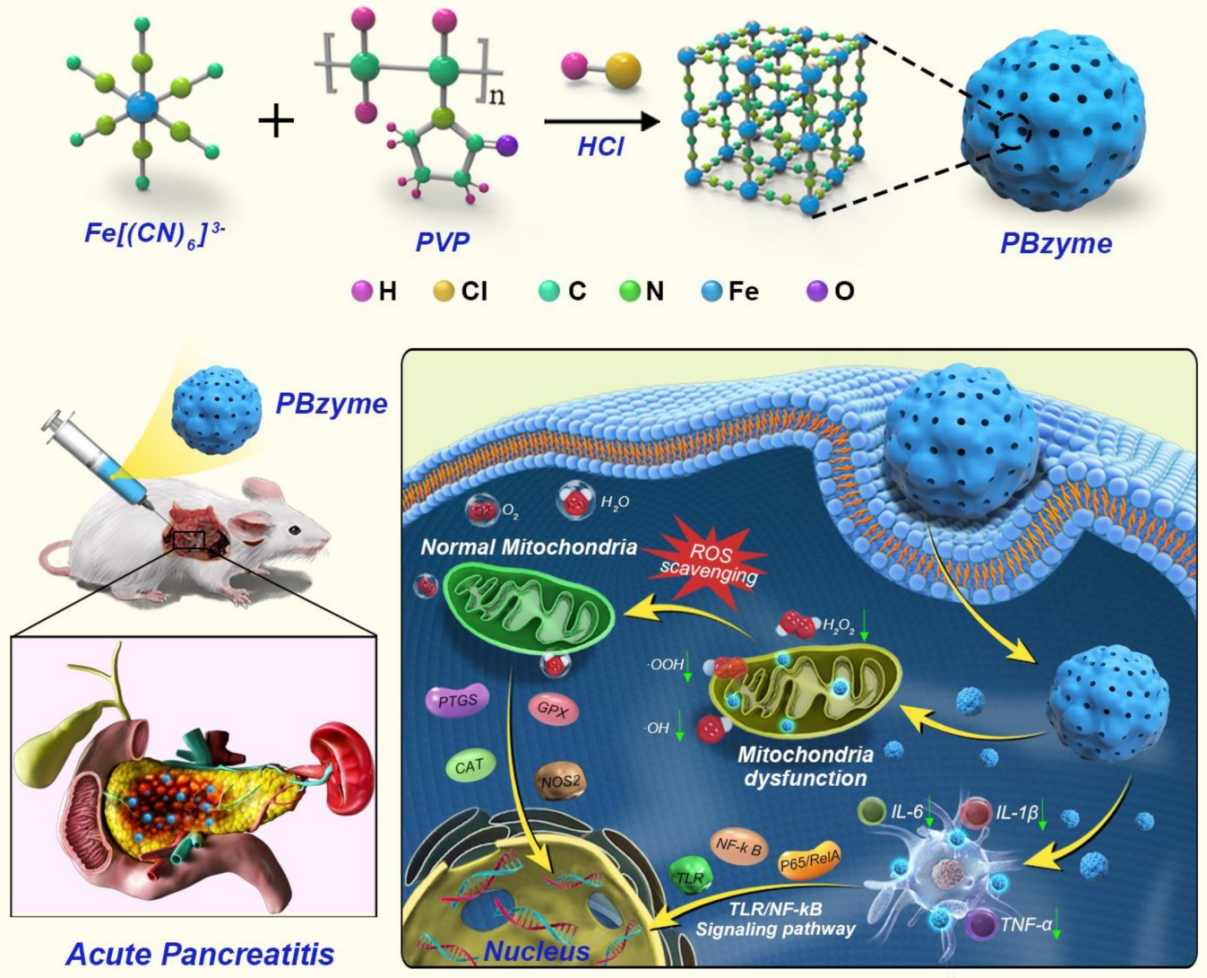
Schematic illustration of therapeutic mechanism of PBzyme prophylactically treats acute pancreatitis by inhibiting activation of the TLRs/NF-κB signaling pathway related to inflammation and oxidative stress. PBzyme affected the expression of Rela, NF-κB, TNF-α, IL-1, and TLR genes by inhibiting activation of the TLR / NF-κB signaling pathway. In addition, PBzyme can scavenge ROS including •OH, •OOH, and H_2_O_2_, and influence the oxidative stress-related genes such as SOD, GPX, PTGS, NOS_2_, and CAT, reducing the apoptosis and necrosis of cells, DNA damage, phospholipid peroxidation and protein oxidation in acute pancreatitis.

Herein, the mechanism of PBzyme with inherent antioxidant and anti-inflammatory bioactivities in the prophylactic treatment of AP was revealed. Large-scale synthesis of monodisperse PBzyme with good biosafety was achieved, satisfying pre-clinical requirements. As illustrated in **Scheme 1**, PBzyme showed good prophylactic therapeutic efficacy in AP. The polymerase chain reaction (PCR) array results indicated that PBzyme had a significant regulatory effect on anti-oxidation and anti-inflammatory proteins. PBzyme inhibited the activation of toll-like receptors (TLRs)/NF-κB signaling pathway related to inflammation and oxidative stress, thereby playing an anti-inflammatory role. Furthermore, PBzyme scavenged hydroxyl radicals, superoxide anion, hydrogen peroxide, and other ROS at the site of AP, thereby reducing cell apoptosis and necrosis, DNA damage, phospholipid peroxidation, and protein oxidation. This study provides a valuable foundation for drug development and prophylactic treatment of AP.

## Materials and methods

### Materials

Polyvinylpyrrolidone (PVP), potassium ferricyanide (K_3_[Fe(CN)_6_]) and hydrochloric acid (HCl) were purchased from Sinopharm chemical Reagent Co., Ltd.

### Preparation of PBzyme

PVP (125 g) and K_3_[Fe(CN)_6_] (12.375 g) were dissolved in HCl (1 M, 1000 mL). The clarified solution was obtained by magnetic stirring for 40 min, and then placed in an oven at 60 ℃ for 16 h, and PBzyme was obtained by centrifugation, separation, and washing with distilled water thrice.

### Characterization of PBzyme

The microstructure and physical characteristics of PBzyme were observed using a JEM-2100F transmission electron microscope (TEM), and scanning electron microscope (SEM). The crystal properties were tested using X-ray diffraction (XRD) (Rigaku D/MAX-2200PX). The chemical status and chemical bonds were characterized using X-ray photoelectron spectroscopy (XPS, ESCAlab250 instrument) and Fourier transform infrared spectroscopy (FTIR) respectively. The specific surface area (SSA) and the pore diameter of PBzyme are tested using N_2_ absorption-desorption technique.The size of PBzyme was measured using dynamic light scattering (DLS, Nano ZS90 Zetasizer, Malvern). The absorption spectrum was recorded using a Shimadzu UV-3600 ultraviolet-visible spectrophotometer.

### Scavenging ability of PBzyme on hydroxylradical (•OH)

The electron spin resonance (ESR) was used to detect the spectra of •OH using a TiO_2_/UV system. TiO_2_ can generate •OH at a wavelength of 340 nm ultraviolet light. Different concentrations of PBzyme (10, 20, and 30 μg/mL) were added to TiO_2_ (0.1 mg/mL) and 50 mM BMPO (cyclic nitrone spin trap), respectively. The ESR spectra were recorded under the following conditions: 20 mW microwave power, 1 G field modulation, and 200 G scanning width.

### Scavenging ability of PBzyme on superoxide anion (•OOH)

1 mM xanthine and 0.2 U/mL xanthine oxidase were mixed for 1.5 min, and BMPO was spin-adducted with superoxide to form the adduct BMPO/•OOH. Different concentrations of PBzyme (10, 20, 30 μg/mL) were added to the xanthine/xanthine oxidase system and detected using a Bruker EMX spectrometer.

### Scavenging ability of PBzyme on hydrogen peroxide (H_2_O_2_)

The ESR spin label oximetry method was used to evaluate the CAT-like mimetics of PBzyme. The following reagents were used: 25 mM H_2_O_2_ solution, and 0.1 mM of 3-carbamoyl-2,2,5,5-tetramethyl-3-pyrroline-1-yloxyl (CTPO) mixed in PBS buffer (pH7.4). All sample solutions were deoxygenated with nitrogen for 10 min before adding H_2_O_2_. Next, different concentrations of PBzyme solution (10, 20, 30 μg/mL) were added to aforementioned solution. 25 mM H_2_O_2_, 0.1 mM CTPO and PBzyme solutions (30 μg/mL) were mixed to the record ESR spectrum at different reaction times (0, 2, 10, 15 and 30 min).The O_2_ concentration in each sample was determined by measuring the K parameter. The actual O_2_ concentration was measured by the following equation: 10^4^[O_2_] = 2.30 - 7.46K.

### Calculation of Michaelis-Menten kinetics

The maximal velocity of the reaction (V_max_) and Michaelis-Menten constant (K_m_) can be calculated as follows:


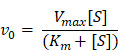



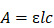


Where *v*_0_ is the initial velocity of the reaction. [S] is the substrate (H_2_O_2_) concentration.

A is the absorbance. ɛ =39000 M^-1^cm^-1^.
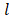
=10 mm. c is the molar concentration.

### Cell culture and *in vitro* cytotoxicity assay

Pancreatic acinar cell line AR42J cells were purchased from the cell bank of the Chinese Academy of Sciences. The cytotoxicity of PBzyme *in vitro* was determined using a standard CCK-8 cell activity assay. AR42J cells were cultured overnight in a 96-well plate (1 × 10^ 4^ cells per well) to adhere to the wall. Different concentrations of PBzyme (0, 12.5, 25, 50, 100, 200 and 400 μg/mL) were added to the well with 100 μL/well.The cells were incubated for 12, 24, and 48 h. Next, the cell survival rate was detected using the standard CCK-8 method (100 μL, V_CCK8_: V_DMEM_ = 1:9). After 40 minutes, the cell survival rate was measured using a micro board reader at a wavelength of 450 nm.

### Animals

The animal experiments were investigated based on the Animal Care Committee of Changhai hospital for AP Research. Balb/c mice were purchased from Shanghai Laboratory Animal Center.

### PBzyme scavenges ROS and inhibits inflammatory cytokines

AR42J cells were randomly divided into the following six groups: control, PBzyme-L (100 μg/mL), PBzyme-H (200 μg/mL), Caerulein, Caerulein+PBzyme-L (100 μg/mL), and Caerulein+PBzyme-H (200 μg/mL) groups. AR42J cells were stimulated by caerulein for 4 h after PBzyme pretreatment. The supernatant was collected to detect the changes of serum amylase (AMS) activity in the supernatant using an amylase activity detection kit. The concentration of pro-inflammatory cytokines was measured by enzyme-linked immunosorbent assay (ELISA) kit. The intensity of ROS in different treatment groups was detected using an flow cytometry.

### Efficacy of PBzyme in the treatment of caerulein-mediated AP

ICR mice were randomly divided into the following four groups: (a) control, (b) Caerulein, (c) Caerulein+PBzyme-L (10 mg/kg), and (d) Caerulein+PBzyme-H (20 mg/kg) groups 30. Mice were allowed free access to water and fasted for 12 h. When establishing the mouse AP model, the optimal dose of caerulein was injected intraperitoneally (0.2 mL) 12 times. Before the second and after the eighth caerulein injection, mice in the Caerulein+PBzyme-L and Caerulein+PBzyme-H groups were injected with PBzyme. The same volume of 0.9% NaCl solution was injected in the control groups. Peripheral blood and tissue samples were collected after 48 h. The concentrations of amylase, TNF-α, IL-1 β, IL-6, and lipase in the serum of mice were detected by ELISA. The pancreatic tissue homogenate was used to measure total protein, the pancreatic tissue oxidative stress indices malondialdehyde (MDA), SOD, glutathione peroxidase (GSH), monocyte chemotactic protein 1 (MCP-1), and inflammatory factor indices IL-1β and IL-6. Pancreatic inflammation was evaluated by hematoxylin and eosin (H&E) staining and pancreatic apoptosis was evaluated by terminal deoxynucleotidyl transferase dUTP nick end labeling (TUNEL) fluorescence staining. The pancreatic histopathological score index in each group was calculated by the Schmidt pancreatic histopathological score standard.

### PCR array analysis

Three samples in each group were used in the experiment. Eighty-seven key genes related to inflammation and oxidation signaling pathways (**Table S1**) were selected for high-throughput PCR array sequencing. The primer design and synthesis prepared by Invitrogen (Carlsbad, CA, USA).

### mRNA extraction

The mRNA extraction kit was used according to the instructions (Qiagen, Hilden, Germany): firstly, 30 mg tissue was weighed and added to 600 µL RLT buffer, and the tissue was crushed using an electric homogenizer. After centrifugation, the supernatant was absorbed and added to 600 µL 70% ethanol. The sample (700 µL) was absorbed onto an Rneasy spin column, and the effluent was discarded by centrifugation. Next, 700 µL Buffer RW1 was added to an Rneasy spin column and centrifuged for 1 min. After centrifugation twice after adding 500 µL RPE buffer to an Rneasy spin column, the Rneasy spin column was placed in a 2 mL tube and centrifuged for 1 min. Finally, the Rneasy spin column was placed in a 1.5 mL tube, and 15 µL Rnase-Free water was added and centrifuged for 1 min to obtain the RNA solution.

### Synthesis of cDNA template by reverse transcription

The 100 μL reverse transcription system was prepared by mixing the following reagents: 8 μL sample RNA, 4 μL oligo (dT) 18 primer, 4 μL reverse transcriptase, 20 μL reverse transcription buffer, 5 μL 10 mM dNTPmix, 1.5 μL RNA enzyme inhibitor, and 56.15 μL DEPC water, and subsequently reverse-transcribed into cDNA.

### PCR array

The RT-PCR dye premix provided by BioTNT (Shanghai, China) was melted and mixed on ice, and 100 µL cDNA sample was mixed with 1 mL PCR water and 1 mL premix to prepare the PCR mixture. The reaction solution (20 μL) was added to each reaction tube, 87 target genes and one reference gene were selected, and RT-PCR experiments were carried out in a qPCR instrument (ViiA 7 Thermo Fisher Science). The RT-PCR results were analyzed using the ViiA 7 software, and the 2^-ΔΔCt^ value was calculated as the relative expression level of the target gene.

There were 3 samples in each group, and the average and standard deviation were used in data processing. Finally, the results of the gene heat map are shown after taking the average.

### Western-blotting analysis

The tissue (100 mg) was ground into powder and 1 mL RIPA lysis buffer was added. After centrifugation, equal concentrations of supernatants were used from each group, and the protein sample was prepared for western blotting analysis. The protein sample was separated by gel electrophoresis and then transferred to membrane. After rinsing with deionized water and PBST, the membrane was immersed in sealing solution and shaken slowly for approximately 1 h. The first antibody diluent containing the antibody against the target protein (NF-κB/p50, NF-κB/p65, TLR4, TLR5, MYD88, Proteintech, USA) was added, sealed at room temperature for 1 h, and then incubated overnight at 4 °C. After washing with TBST thrice, it was transferred to a solution containing horseradish peroxidase, diluted and incubated for 1 h. The A and B luminescence solutions in ECL hypersensitive chemiluminescence reagents were diluted and mixed, each strip was dripped with 0.2 mL, and imaged using an Amersham Imager 600 (GE Healthcare, Chicago, IL USA).

### Biosafety evaluation of PBzyme

ICR mice were randomly divided into the following five groups: control group, PBzyme group (n = 5). After fasting for 12 h, each mouse was administered with Pbzyme (20 mg/kg) by intraperitoneal injection. The mice were anesthetized with ether after one and 30 days of injection, and eyeball blood was collected in an anticoagulant tube containing ethylene diamine tetraacetic acid. Routine blood examination and cell counting were performed. After 30 min, the whole blood of the mice was centrifuged at 7000 × g for 15 min. The supernatant was collected and detected using an ELISA kit for blood biochemical related index. The mice were dissected, and the main organs were fixed and analyzed by H&E staining.

### Pharmacokinetic study

Mice were injected intraperitoneally with 200 μL PBzyme. Approximately 10 μL blood was collected at specified intervals (0, 0.17, 0.3, 0.5, 1, 2, 4, 6, 12, 24, and 48 h), and then diluted with aqua regia. The concentration of Fe was quantified by inductively coupled plasma mass spectrometry (ICP-MS), and the half-life of blood circulation (t_1/2_) was calculated.

### *In vivo* biodistribution

For biodistribution analysis, the mice were euthanized at different time points (2 h, 1 d, 2 d, and 30 d) after intraperitoneal injection of 200 μL PBzyme. The main organs (liver, heart, lung, spleen, kidney, and pancreas) were collected, weighed, and then digested with aqua regia. ICP-MS was performed to quantitatively organize the elements of Fe.

### Data statistics and analysis

All data are expressed as mean ± standard deviation (SD). ORIGIN software and GRAPHPAD software platform were used to compare the experimental data, t-test was used to compare the mean of the two groups, one-way ANOVA was used to analyze the three groups and above, and Schmidt pancreatic histopathological score was analyzed by repeated measurement analysis of variance of the general linear model in SPSS software. (*, p < 0.05; **, p < 0.01; ***, p < 0.001, ****, p < 0.0001).

## Results and Discussion

### Construction and characteristics of PBzyme

**Figure 1:**
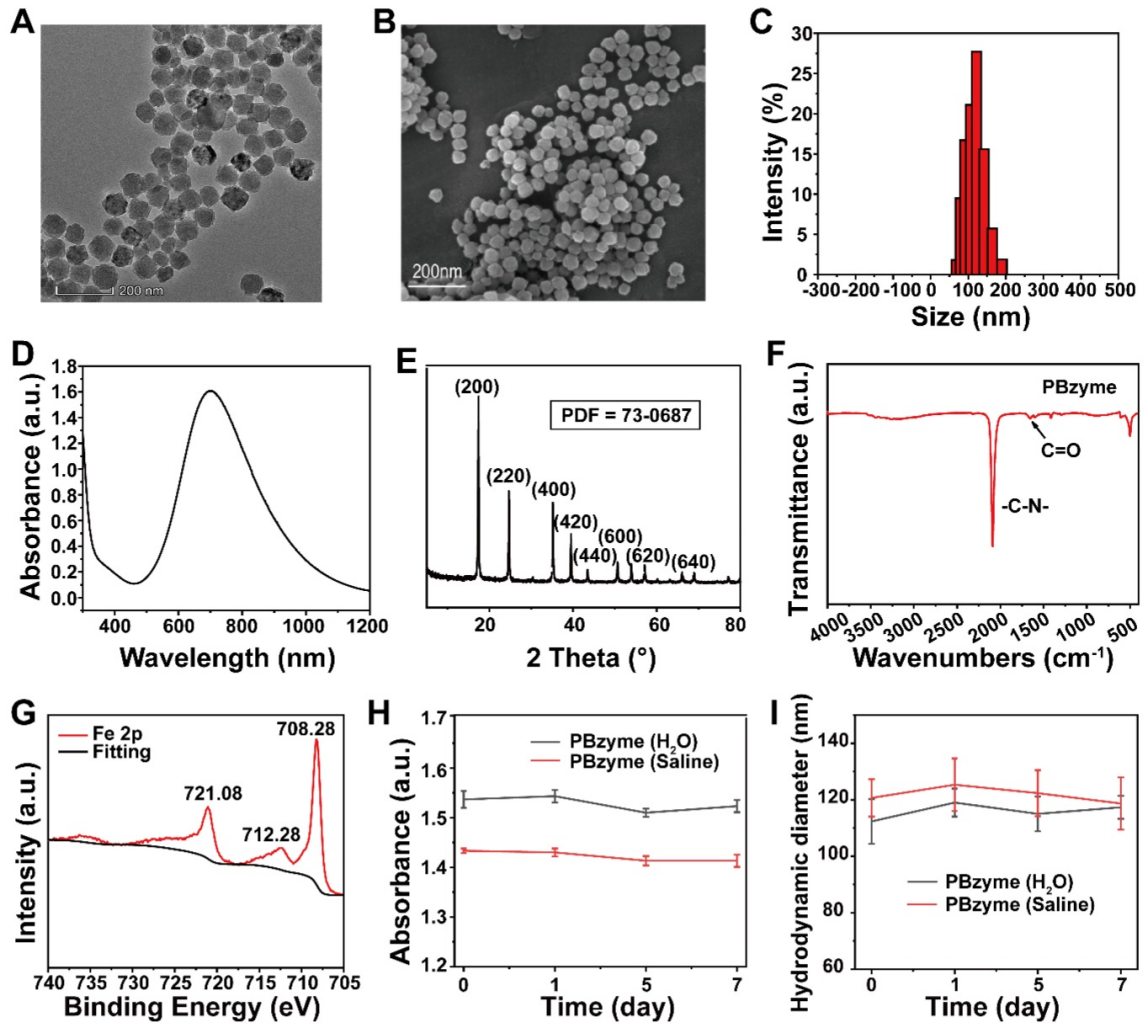
Synthesis and characterization of PBzyme. (A) TEM image of PBzyme; scale bar: 200 nm. (B) SEM image of PBzyme; scale bar: 200 nm. (C) Dynamic light scattering of PBzyme dispersed in aqueous solution. (D) The UV-vis spectra of PBzyme dispersed in aqueous solution. (E) XRD patterns of PBzyme. (F) FTIR spectra of PBzyme.(G) XPS spectra of PBzyme in the Fe 2p region. (H) Time-dependent UV-vis absorbance changes of PBzyme in water and saline. (I) Time-dependent hydrodynamic diameters changes of PBzyme in water and saline.

The necessary features for preclinical trials of nanodrugs include controlled preparation at the gram level and good dispersion stability under physiological conditions. Various PB nanoparticles with different structures have been prepared, and these have exhibited good dispersion stability under physiological conditions 34. However, the controlled mass production of PB nanoparticles even at the gram level remains a challenge. Based on our previous research 32, by optimizing the preparation process of PBzyme, we achieved mass production of PBzyme at the gram level, thereby demonstrating excellent potential for clinical translation. The ratio of PVP plays an important role in its optimization. We modified the synthesis of PBzyme via changing the ratio of PVP and iron source with 1 M HCl. The mass of iron source and PVP can be increasing to 12.375 and 125 g, respectively. And more than 5 g PBzyme was obtained in one preparation. The structure, morphology, and size of PBzyme was observed by TEM (**Figure 1A, Figure S1A-C**) and SEM (**Figure 1B**). PBzyme displayed a uniform sphere-like structure with a ~60-nm average diameter and ~110-nm average hydrodynamic size (**Figure 1C**). The prepared PBzyme displayed a characteristic absorbance peak at 700 nm caused by electron transition in Fe^II^-C≡N-Fe^III^ (**Figure 1D**). X-ray powder diffraction showed that PBzyme has various characteristic peak diffraction surfaces including 17.4^°^ (200), 24.5^°^ (220), 35.2^°^ (400), and 39.5^°^ (420) (**Figure 1E**). Fourier transform infrared spectroscopy displayed a characteristic peak at 2085 cm^-1^ for Fe^II^-C≡N-Fe^III^ in PBzyme (**Figure 1F**). X-ray photoelectron spectroscopy (XPS) displayed the chemical state of elements in PBzyme. The peaks of Fe2p^3/2^ (712.28 eV) and Fe2p^1/2^ (721.08 eV) matched that of Fe^III^ in Fe_3_[Fe_2_(CN)_6_]_4_, and the peak at 708.28 eV revealed the existence of Fe2p^3/2^ in [Fe_2_(CN)_6_]^4-^ (**Figure 1G**). The specific surface area (SSA) and the pore diameter of PBzyme were tested by N_2_ absorption-desorption technique with Brunauer-Emmett-Teller. The results showed that the specific surface area of PBzyme is 105 m^2^ g^ -1^ and the average pore diameter is 11.7 nm (**Figure S2**). During the observation of PBzyme dispersed in water and normal saline for seven days, there was no significant change in the UV-vis absorbance or hydrodynamic diameters, indicating that PBzyme is stable in saline and can be preserved long-term (**Figure 1H-I, Figure S3-4**).

### Intrinsic ROS scavenging properties of PBzyme

**Figure 2:**
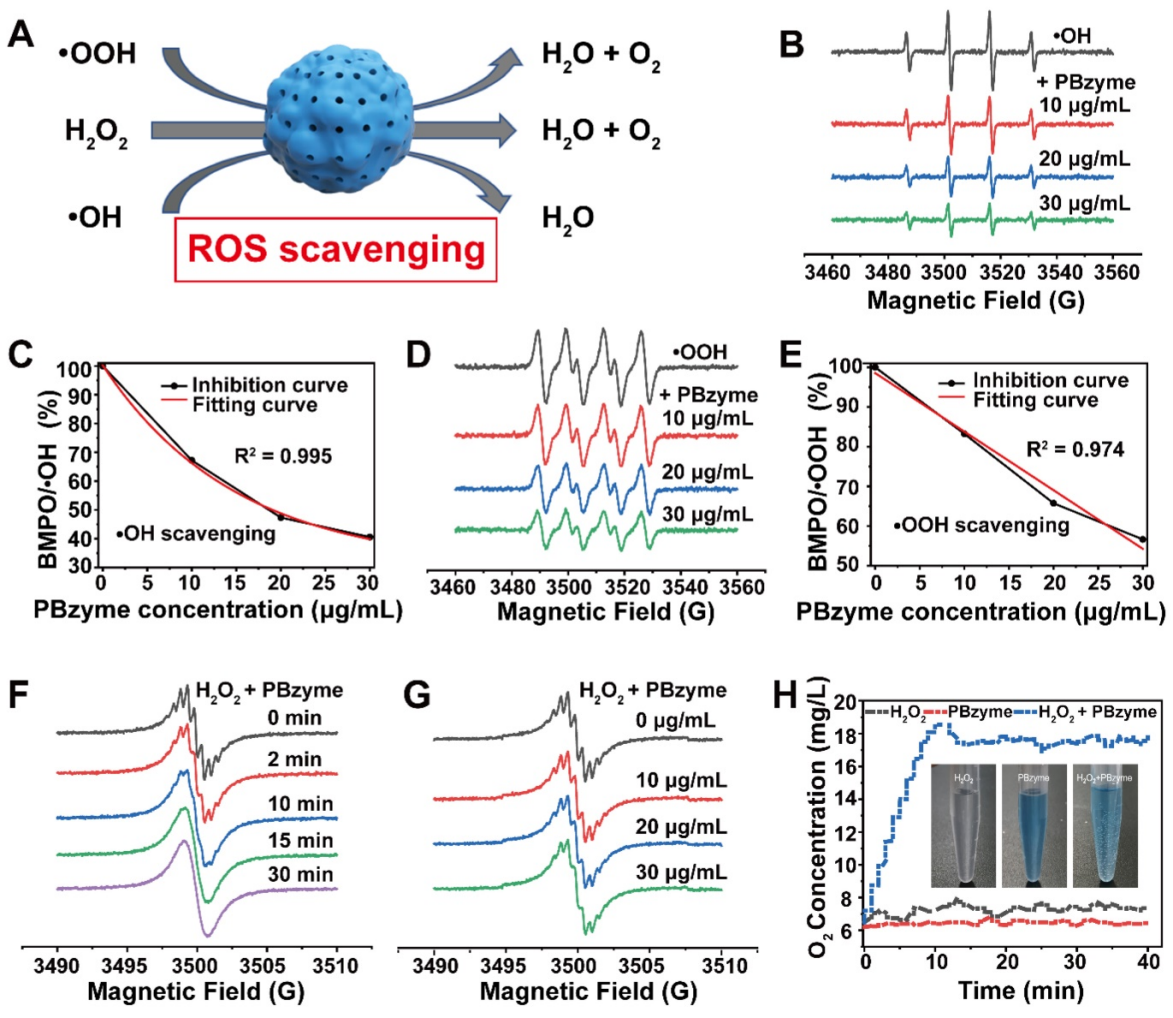
The scavenging effect of PBzyme on reactive oxygen species *in vitro*. (A) PBzyme with ROS scavenging property converted toxic ROS including •OH, •OOH, and H_2_O_2_ into non-toxic H_2_O and O_2_. (B) The scavenging effect of PBzyme on •OH generated from a TiO_2_/UV system (pH 5.5). (C) Quantitative analysis of inhibition rate of •OH in different concentrations of PBzyme *in vitro*. (D) The scavenging effect of PBzyme on •OOH (pH 5.5). (E) Quantitative analysis of inhibition rate of •OOH in different concentrations of PBzyme *in vitro*. The effect of PBzyme on the generation of O_2_ from H_2_O_2_ determined by ESR oximetry. (F) O_2_ generation at different reaction time (0, 2, 10, 15, and 30 min) (pH 7.4). (G) O_2_ generation from H_2_O_2_ degradation catalyzed by PBzyme was measured in a closed chamber with a mixture of samples containing 0.1 mM CTPO, 25 mM H_2_O_2_ and PBzyme (0, 10, 20, 30 μg/mL) in PBS buffer (pH 7.4). (H) O_2_ generation was measured by the dissolved oxygen meter. Illustration: Images of different systems reacting for 15 min at pH 7.4.

•OH, •OOH, and H_2_O_2_ were selected as the main ROS models to investigate the ROS scavenging properties of PBzyme (**Figure 2A**). The ROS scavenging properties of PBzyme is dependent on the structure, size, specific surface, concentration and so on. The ESR results showed that the characteristic peak intensity of BMPO/•OH and BMPO/•OOH decreased with the increasing PBzyme concentrations. The •OH scavenging efficiency of PBzyme was 32.6% (10 μg/mL), 52.8% (20 μg/mL), and 59.1% (30 μg/mL), fitting the curve relationship between •OH scavenging rate and PBzyme concentration. This relationship was reflected by ESR intensity and showed good non-linear correlation with an R^2^ value of approximately ~ 0.995. (**Figure 2B-C**). The •OOH scavenging efficiency was 16.8% (10 μg/mL), 34.2% (20 μg/mL), and 41.9% (30 μg/mL), fitting the curve relationship between the •OOH scavenging rate and PBzyme concentration, and thus revealed a good linear correlation with an R^2^ value of approximately ~0.974 (**Figure 2D-E**). We conducted ESR oximetry to determine the effect of PBzyme on the generation of O_2_ from H_2_O_2_. CTPO was selected to investigate the generation of O_2_ because CTPO is highly sensitive to changes in O_2_ (**Figure S5**). The ESR spectrum showed that the O_2_ concentration increased with a certain range of increasing PBzyme concentrations; O_2_ generation also increased with increasing reaction times (**Figure 2F-G**). Furthermore, a dissolved oxygen meter was used to detect O_2_ generated from H_2_O_2_ catalyzed by PBzyme. Neither H_2_O_2_ nor PBzyme alone increased the dissolved amount of O_2_. The O_2_ concentration curve of the H_2_O_2_ and PBzyme reaction system was observed to be clearly increased in 10 min. A large number of bubbles were formed in the H_2_O_2_ and PBzyme reaction system, reflecting that PBzyme catalyzed the conversion of H_2_O_2_ to H_2_O and O_2_ (**Figure 2H**), and the production of O_2_ increased with increasing pH (**Figure S6**). In addition, the catalytic kinetics of PBzyme were conducted. According to the Michaelis-Menten kinetic property of the nano-catalytic system, the catalytic performance of PBzyme was evaluated. Hydrogen peroxide (1.25, 2.5, 5, 10, 20 mM) , PBzyme (10 μg/mL) was selected. The average initial velocity (*v*_0_) is calculated based on the time-course absorbance (**Figure S7A**). According to Beer-Lambert 's law, the change in absorbance will be converted into *v*_0_ 30. Then, the relationship between *v*_0_ and the corresponding concentration was fitted by Michaelis-Menten curve (**Figure S7B**). In addition, a linear double reciprocal curve for determining the Michaelis constant (*Km*) and the maximum velocity (*V*max) is obtained (**Figure S7C**). The *K*m of PBzyme was calculated as 7.825 mM,* V*max was calculated as 6.378 × 10^-7^ Ms^-1^. Overall, these results demonstrate that PBzyme can robustly scavenge ROS by converting harmful ROS into harmless H_2_O and O_2_.

### Intrinsic anti-oxidative and anti-inflammatory bioactivities of PBzyme *in vitro*

**Figure 3:**
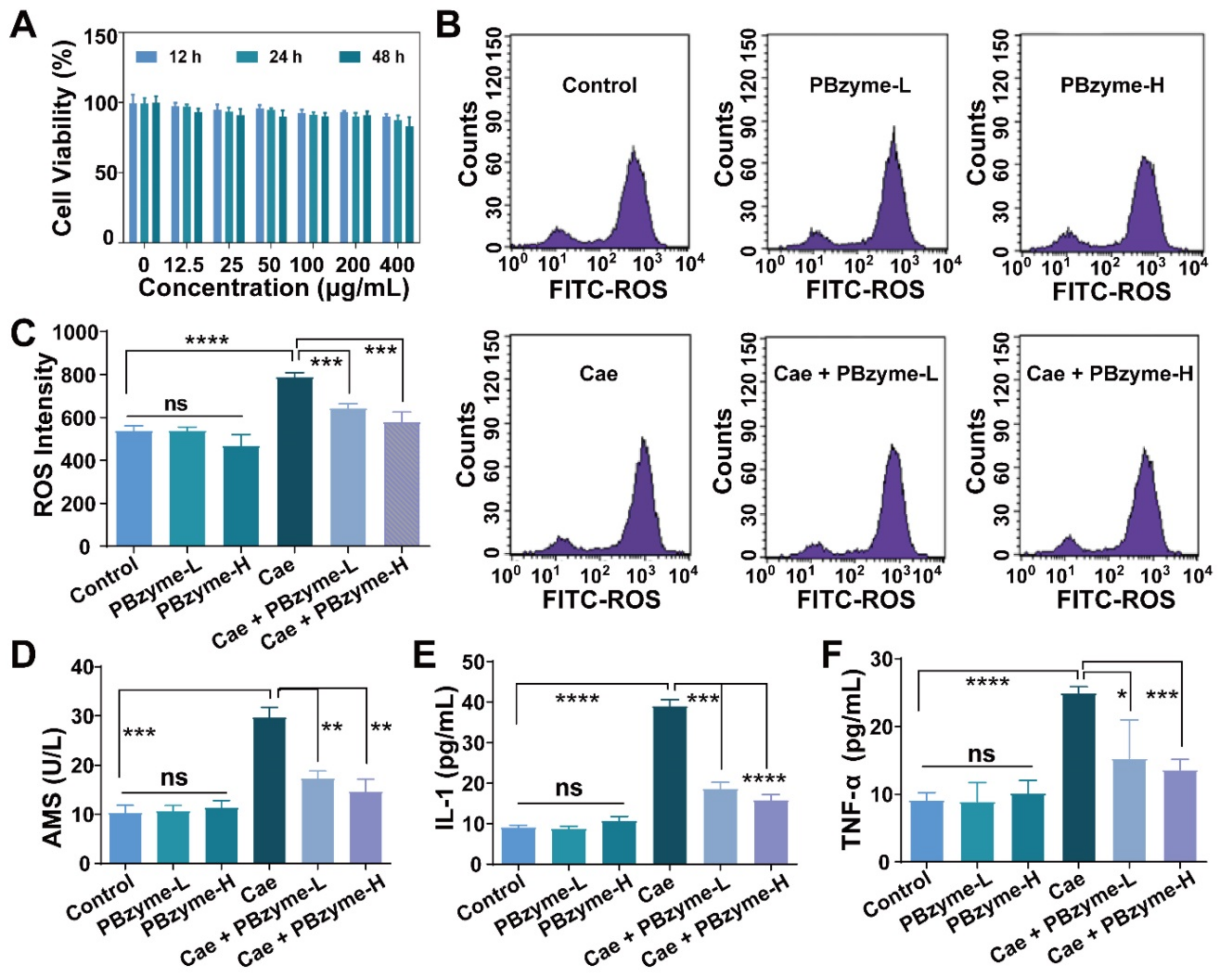
PBzyme scavenges ROS and inhibits inflammatory cytokines. (A) AR42J cell viability co-cultured with different concentrations of PBzyme for 12, 24, and 48 h; (B) ROS intensity changes in different treatment groups by flow cytometry; (C) Quantitative analysis of ROS intensity. Changes of Serum amylase and inflammatory factors after different treatment of caerulein and PBzyme. (D) AMS, (E) IL-1 and (F) TNF-α. (*, p < 0.05; **, p < 0.01; ***, p < 0.001; ****, p < 0.0001).

Based on the excellent catalytic properties of PBzyme on ROS, we examined its efficacy at the cellular level. There was no significant difference in the cell survival rate compared to that in the control group within the concentration of 400 μg/mL, indicating that PBzyme had good cytocompatibility at the experimental concentration (**Figure 3A**). Subsequently, we investigated the changes in ROS expression and inflammatory cytokines in caerulein-stimulated AR42J cells pretreated with PBzyme. Here, 100 μg/mL and 200 μg/mL were chosen for *in vitro* and *in vivo* experiment referring to the relevant literature 30. The intensity of ROS in various groups was examined by flow cytometry. There was no significant difference among the control, PBzyme-L (100 μg/mL), and PBzyme-H (200 μg/mL) groups. The level of ROS significantly increased in caerulein-stimulated cells without PBzyme pretreatment. However, PBzyme pretreatment scavenged ROS in caerulein-stimulated cells, showing no significant difference compared to that in the control group (**Figure 3B-C**). Some targeted biomarkers in AP, including serum AMS, TNF-α, and IL-1, were investigated in an AR42J cell model. There was no significant difference in these proteins among the control group, PBzyme-L (100 μg/mL) and PBzyme-H (200 μg/mL) groups without caerulein stimulation**.** After caerulein stimulation, the levels of AMS, IL-1, and TNF-α increased from 10.3 to 29.7 U/L, 9.1 to 38.9 pg/mL, and 9.2 to 24.9 pg/mL respectively. However, PBzyme downregulated the levels of AMS, IL-1, and TNF-α to 14.6 U/L, 15.8 pg/mL, and 13.6 pg/mL in caerulein-stimulated cells, respectively (**Figure 3D-F**). Based on these results, PBzyme can effectively eliminate ROS and significantly inhibit the release of inflammatory cytokines in the caerulein-stimulated AP cell model.

### Blood circulation and biodistribution of PBzyme

It is necessary to ensure the effectiveness and biocompatibility of PBzyme *in vivo*. The pharmacokinetic behavior of PBzyme was investigated after its intraperitoneal administration in mice. As shown in** Figure 5F**, the circulation half-life was calculated as 3.03 h. In addition, the biodistribution in major organs and pancreas accumulation of PBzyme at different time points in mice were determined before treatment *in vivo* by ICP-MS. The results showed that PBzyme was mainly concentrated in the liver (approximately 65.02%), and its accumulation in the pancreas was around 10.80% at one day after PBzyme injection; this amount was adequate for the desired therapeutic efficacy (**Figure 5G**).

### *In vivo* therapeutic effect of PBzyme with intrinsic anti-oxidative and anti-inflammatory bioactivities on AP

**Figure 4:**
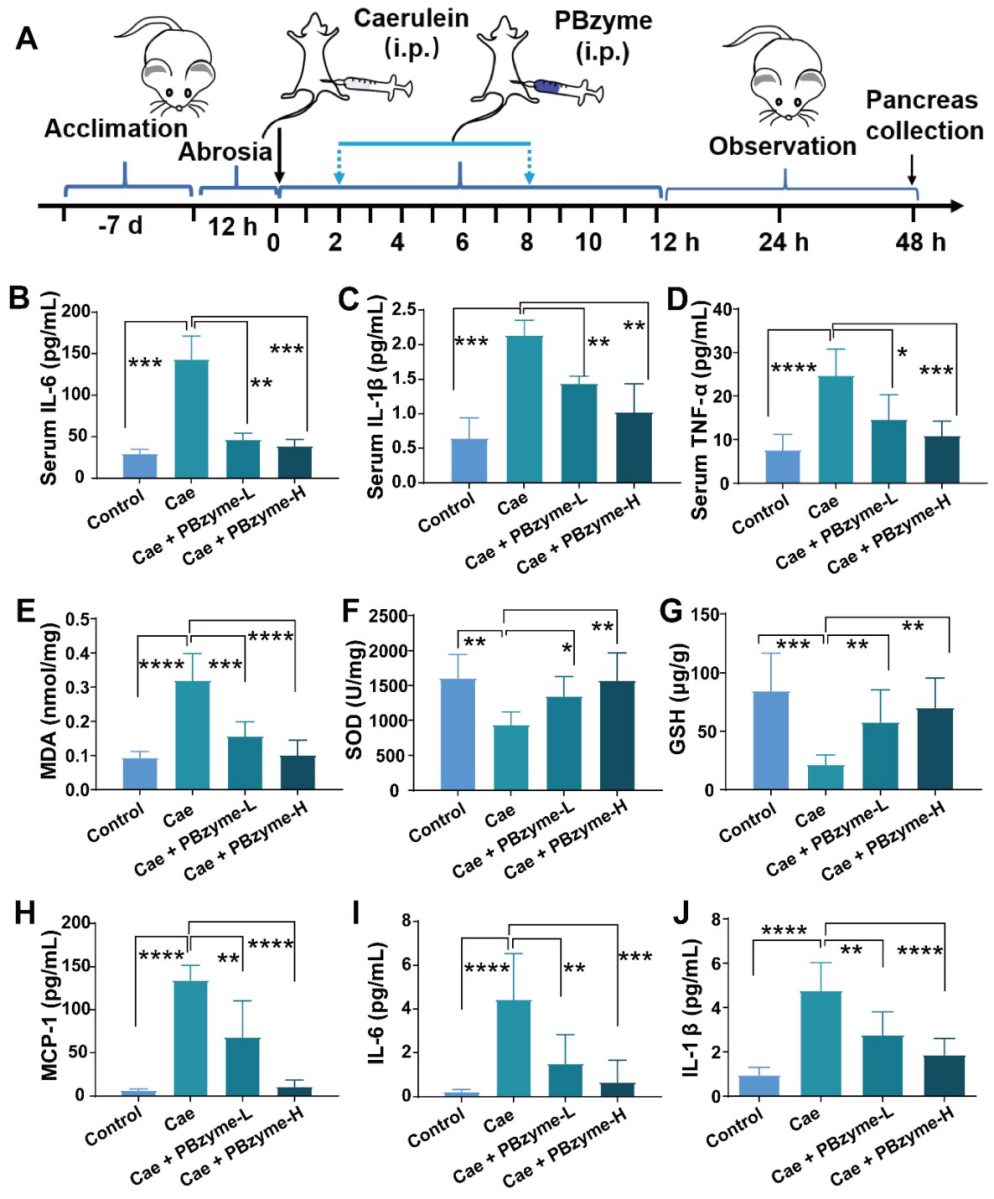
PBzyme pretreatment can prophylactically treat AP by inhibiting the release of inflammatory factors *in vivo*. (A) Overall experimental procedure. The levels of (B) serum IL-6, (C) serum IL-1β, and (D) serum TNF-α changed in various groups. The indexes of pancreatic tissue oxidation and inflammation were evaluated after the mice treated with different experimental groups. The levels of (E) MDA, (F) SOD, (G) GSH, (H) MCP-1, (I) IL-6, and (J) IL-1β changed in various groups. (*, p < 0.05; **, p < 0.01; ***, p < 0.001; ****, p < 0.0001).

Subsequently, we investigated the *in vivo* preventive therapeutic effect of PBzyme on AP. Intraperitoneal injection of caerulein has become the most widely used experimental animal model of AP because of its easy induction, non-invasive operation, and good repeatability, and the histopathological features of its pancreas are similar to those of human AP 35. Following PBzyme preventive treatment, the increased level of serum AMS and serum lipase (LPS) reduced caerulein in the AP mouse model, particularly in the Caerulein + PBzyme-H group (**Figure 4**). Additionally, the serum AMS and LPS values in the control and Caerulein + PBzyme-H groups did not significantly differ (**Figure S8**). Inflammatory cytokines (IL-1, IL-6, and TNF-α) play a central role in the progression of AP. Pro-inflammatory cytokines such as IL-6, IL-1β, and TNF-α in the serum can also reflect the severity of AP 6, 36, 37. TNF-α and IL-1β are primary cytokines that enhance the inflammatory response by activating macrophages and regulating the release of other inflammatory mediators. In the AP animal model, TNF-α can cause upregulation of other cytokines, free radical synthesis, cell death and endothelial cell activation. IL-6 is among the best indices for distinguishing mild and severe AP 37, 38. Consistent with the results of cell experiments, PBzyme downregulated the serum levels of IL-6, IL-1β, and TNF-α in a caerulein-induced AP mouse model (**Figure 4B-D**), indicating the preventive therapeutic effect of PBzyme on AP.

The levels of ROS, including MDA and xanthine oxidase, as well as oxidative stress markers, such as myeloperoxidase, are increased in AP. MDA is not only an important parameter for indicating the antioxidant capacity of the body, but also indirectly reflects the degree of tissue peroxidation damage 13. The concentration of MDA in pancreatic tissue in the Caerulein group was significantly higher than that in the control group, demonstrating serious lipid peroxidation-induced cell damage in the pancreatic tissue. After PBzyme treatment, the concentration of MDA was decreased, and there was no significant difference between the Caerulein + PBzyme-H group and the control groups (**Figure 4E**). SOD and GSH can scavenge harmful free radicals to relieve damage caused by the oxidation of free radicals in the body. GSH is another reliable indicator of oxidative stress in cells. Depletion of GSH leads to early activation of digestive enzymes in acinar cells, thus triggering the inflammatory process. Changes in the levels of SOD and GSH can disrupt the balance of the redox state in cells, causing oxidative damage to cells 36, 39. In the Caerulein group, the levels of SOD and GSH in the pancreatic tissue were significantly decreased, indicating oxidative cell damage and inflammation. PBzyme, through its powerful antioxidative and anti-inflammatory effects, significantly increased the levels of SOD and GSH in the caerulein-induced AP mouse model (**Figure 4F-G**). MCP-1 is also an important proinflammatory cytokine. The levels of proinflammatory cytokines, such as MCP-1, IL-6, and IL-1β in the pancreatic tissue of the Caerulein + PBzyme-L and Caerulein+PBzyme-H groups were notably lower than those of the Caerulein group, and similar to those in the control group (**Figure 4H-J**). PBzyme can drive the intrinsic anti-inflammatory bioactivity of these cytokines to significantly alleviate inflammation in a caerulein-induced AP mouse model.

**Figure 5:**
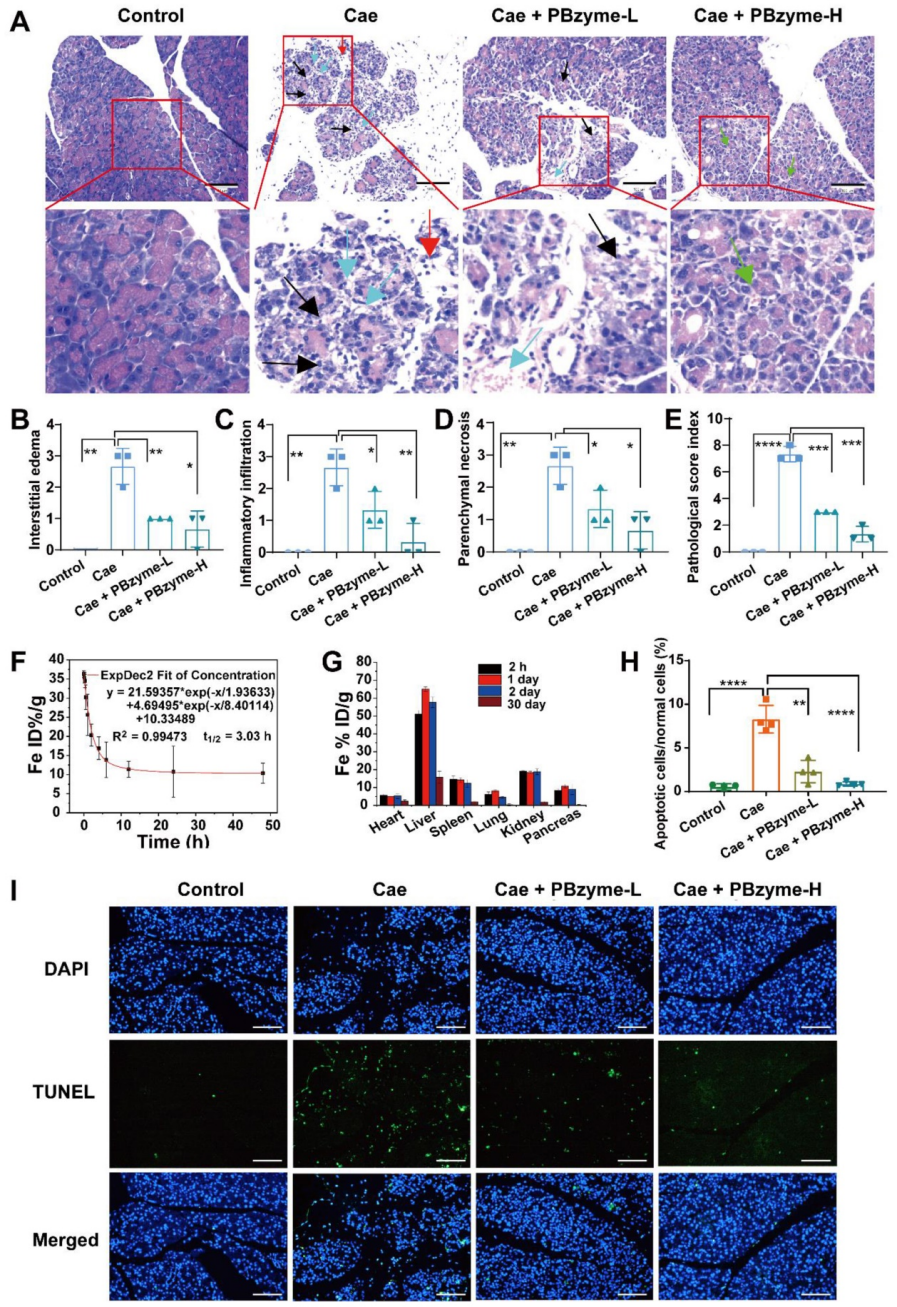
The histopathological changes of pancreas were analyzed by H&E staining and TUNEL apoptosis analysis to evaluate the efficacy of PBzyme after the mice were treated in different experimental groups. (A) H&E staining of pancreatic tissue. (Control group: The pancreatic glands were arranged densely and the structure was normal. Cae group:The black arrow showed that the pancreatic gland was significantly reduced, part of the gland was destroyed, the stroma was significantly widened, loose, and edema, there were more inflammatory cells, mainly lymphocytes and a small amount of neutrophils infiltration, and interstitial vascular hyperplasia and dilatation. The blue arrow showed the proliferation and dilatation of blood vessels in the stroma. The red arrow showed neutrophil infiltration. Cae + PBzyme-L group: The black arrow indicated a slight reduction of glands and destruction of individual glands and the blue arrow indicated dilated blood vessels. Cae + PBzyme-H group: The pancreatic glands were arranged densely, the structure was normal, the stroma was not significantly broadened, and a small number of small vessels could be seen in the stroma.The green arrow indicated the small blood vessels in the interstitium (no obvious hyperplasia and dilatation). Scale bar:100 μm. Schmidt Pancreatic histopathological score. (B) Interstitial edema histopathological score, (C) Inflammatory infiltration histopathological score, (D) Parenchymal necrosis histopathological score, (E) Schmidt Pancreatic histopathological total score. (F) The blood circulation time of intraperitoneal administration of PBzyme was measured by ICP-MS. Half-life was calculated as 3.03 h (n=5). (G) Quantitative biodistribution of PBzyme in the main organs of mice determined by ICP-MS at different time points. (H) The quantitative evaluation of Pancreatic apoptosis by TUNEL fluorescence staining. (I) TUNEL fluorescence staining for evaluation of Pancreatic apoptosis. (*, p < 0.05; **, p < 0.01; ***, p < 0.001; ****, p < 0.0001). Scale bar:100 μm.

Histopathological changes in the pancreas in each group were analyzed by H&E staining (**Figure 5A**). Diffuse and localized edema, swelling, and hardening of the pancreas were observed by the naked eye. In H&E staining of the pancreas from the caerulein-induced AP mouse model, we detected interstitial edema of the pancreas and infiltration of neutrophils and lymphocytes in the interstitial and parenchyma, accompanied by hemorrhage and necrosis. According to the Schmidt score standard of pancreatic histopathology in mice, the pancreatic pathology was scored in each group for pancreatic interstitial edema, inflammatory infiltration, parenchyma necrosis, and parenchyma hemorrhage (**Figure S9**). The levels of interstitial edema (**Figure 5B**), inflammatory infiltration (**Figure 5C**), parenchymal necrosis (**Figure 5D**), and the pathological score index (**Figure 5E**) in the Caerulein group were higher than those in the other groups. After PBzyme treatment, particularly in the Caerulein + PBzyme-H group, the pancreas recovered significantly. Interstitial edema, inflammatory infiltration, and parenchymal necrosis of the pancreas in the Caerulein + PBzyme-H group were clearly reduced, and the pathological score index declined to 1.6, which was ascribed to the anti-oxidative and anti-inflammatory bioactivities of PBzyme (**Figure 5B-E**). Apoptosis of the pancreatic tissue was evaluated by TUNEL staining, showing that the number of apoptotic cells in the pancreatic tissue in the Caerulein group was higher than that in the control group. The numbers of apoptotic cells in the Caerulein + PBzyme-L and Caerulein + PBzyme-H groups were decreased compared to that in the Caerulein group (**Figure 5H-I**).

### Probable mechanism of PBzyme ameliorating AP

**Figure 6:**
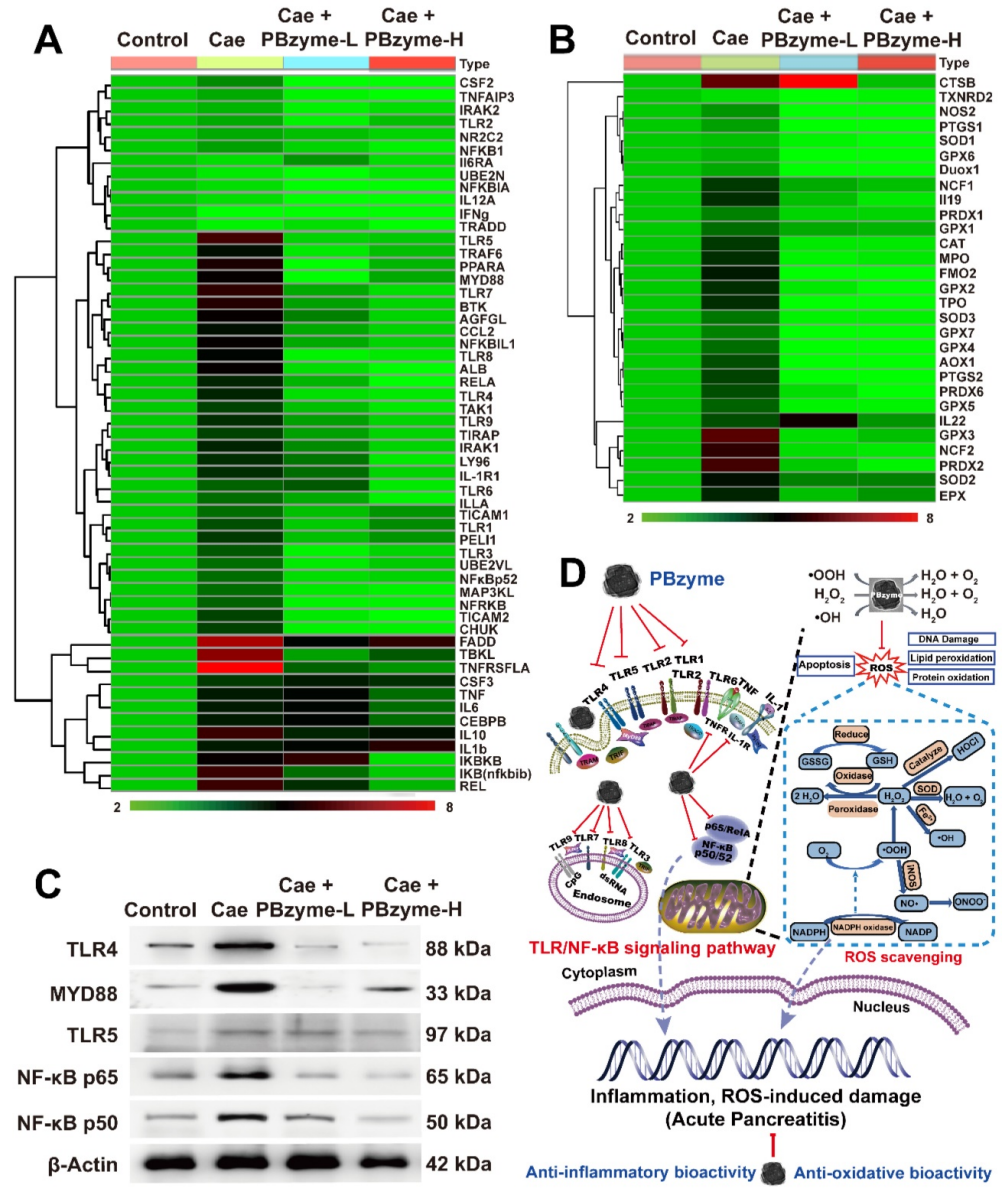
Inflammatory and oxidative stress related genes cluster heat map of the control, Caerulein, Caerulein + PBzyme-L, and Caerulein + PBzyme-H groups. A total of 84 expressed genes most related to inflammation and oxidation were selected to heat map analysis, including 55 genes related to inflammation and 29 genes related to oxidation. (A) inflammation-related genes cluster heat map. (B) oxidative stress related genes cluster heat map. (C) Representative western blotting images of TLR4, MYD88, TLR5, NF-κB**/**65, NF-κB**/**50 related to Toll-like receptor (TLR) and NF-κB signaling pathway in various groups. (n=3) (D) PBzyme affected the expression of TLR genes including *TLR1*,* TLR2*, *TLR3*,* TLR4*,* TLR5*,* TLR6*,* TLR7*,* TLR8*, and* TLR9*, possibly by inhibiting activation of the TLR signaling pathway. In addition, Rela, NF-κB, TNF-α, and IL-1, which are related to activation of NF-κB signaling pathway were strongly inhibited by PBzyme. Furthermore, PBzyme can scavenge ROS including •OH, •OOH, and H_2_O_2_, and influence the oxidative stress-related genes such as* SOD*,* GPX*,* PTGS*,* NOS2*, and* CAT*, reducing the apoptosis and necrosis of cells, DNA damage, phospholipid peroxidation, and protein oxidation in caerulein-induced AP mouse model. Thus, PBzyme may drive intrinsic anti-oxidative and anti-inflammatory activities in the preventive treatment of AP by inhibiting activation of the TLR/NF-κB signaling pathway and scavenging ROS.

We next investigated the probable mechanism of PBzyme and its intrinsic anti-oxidative and anti-inflammatory bioactivities in the preventive treatment of AP via PCR array. Real-time quantitative PCR is the most sensitive and reliable method for detecting gene expression. A total of 87 genes, mostly related to oxidation and inflammation were examined. Among these genes, 79 were upregulated in the Caerulein group, and eight showed no significant change compared to that in the control group (**Figure S10**). PBzyme inhibited the expression of 73 genes upregulated by caerulein, indicating that PBzyme had good therapeutic potential against anti-oxidative and inflammatory stress by regulating related genes. Among the 87 genes evaluated, we selected 84 differentially expressed genes related to inflammation and oxidation for heat map analysis, including 55 genes related to inflammation and 29 genes related to oxidation. Analysis of the cluster heat map revealed a large difference in overall gene expression between the Caerulein and control group. Compared to the control group, most genes in the Caerulein group showed significantly upregulated expression. Following treatment with PBzyme, the upregulated gene expression in the AP mouse model was decreased to levels similar to those in the control group (**Figure 6A-B**). We also performed pathway screening using the Kyoto Encyclopedia of Genes and Genomes (KEGG) database and found that PBzyme inhibited several key inflammatory pathways closely related to the pathogenesis of various inflammatory diseases, including the toll-like receptor (TLR) signaling pathway, NF-κB signaling pathway, and TNF signaling pathway (**Figure S11**). PBzyme affected the expression of TLR genes including *TLR1*,* TLR2*, *TLR3*,* TLR4*,* TLR5*, *TLR6*,* TLR7*,* TLR8*, and* TLR9*, possibly by inhibiting activation of the TLR signaling pathway. In addition, Rela, NF-κB, TNF-α, and IL-1, which are related to the activation of NF-κB signaling pathway, were strongly inhibited by PBzyme (**Figure 6A**). PBzyme also influenced oxidative stress-related genes, such as *SOD*,* GPX*, *PTGS*,* NOS2*, and *CAT* to regulate oxidative stress in the caerulein-induced AP mouse model, which was ascribed to the anti-oxidative bioactivity of PBzyme (**Figure 6B**). Considering the significant role of the protein levels related to the TLR and NF-κB signaling pathways in the biological mechanism of AP and further studies on the mechanism of PBzyme in the preventive treatment of AP, we studied the expression of proteins related to the TLR/NF-κB signal pathway in different groups by western blotting. Expression of the TLR signaling pathway-related proteins, such as TLR4, MYD88, and TLR5 and NF-κB signaling pathway related protein, such as NF-κB**/**p65 40, 41 and NF-κB**/**p50 was examined. Consistent with the results described above, compared with the control group, the expression of TLR4, MYD88, and TLR5 protein in mice with AP stimulated by caerulein was increased, and PBzyme treatment significantly reduced the expression of the TLR4, MYD88, and TLR5 proteins. Western blotting showed that the expression levels of NF-κB**/**p65 and NF-κB**/**p50, which are key proteins involved in NF-κB signaling pathway, were higher in the Caerulein group than in the control group. PBzyme treatment decreased the expression of NF-κB**/**p65 and NF-κB**/**p50 (**Figure 6C**,**Figure S12**). What needs to be explained is that CTSB was up-regulated in Caerulein + PBzyme-L group (**Figure 6B**), but the overall results (inflammatory cytokines, H&E, TUNEL and the protein expression related to TLR and NF-κB signaling pathways) showed the therapeutic effect of PBzyme in AP, whether in the Caerulein + PBzyme-L and Caerulein + PBzyme-H groups. On the whole, PBzyme may drive intrinsic anti-oxidative and anti-inflammatory activities in the preventive treatment of AP by inhibiting activation of the TLR/NF-κB signaling pathway and scavenging ROS (**Figure 6D**).

### *In vivo* biosafety of PBzyme

**Figure 7:**
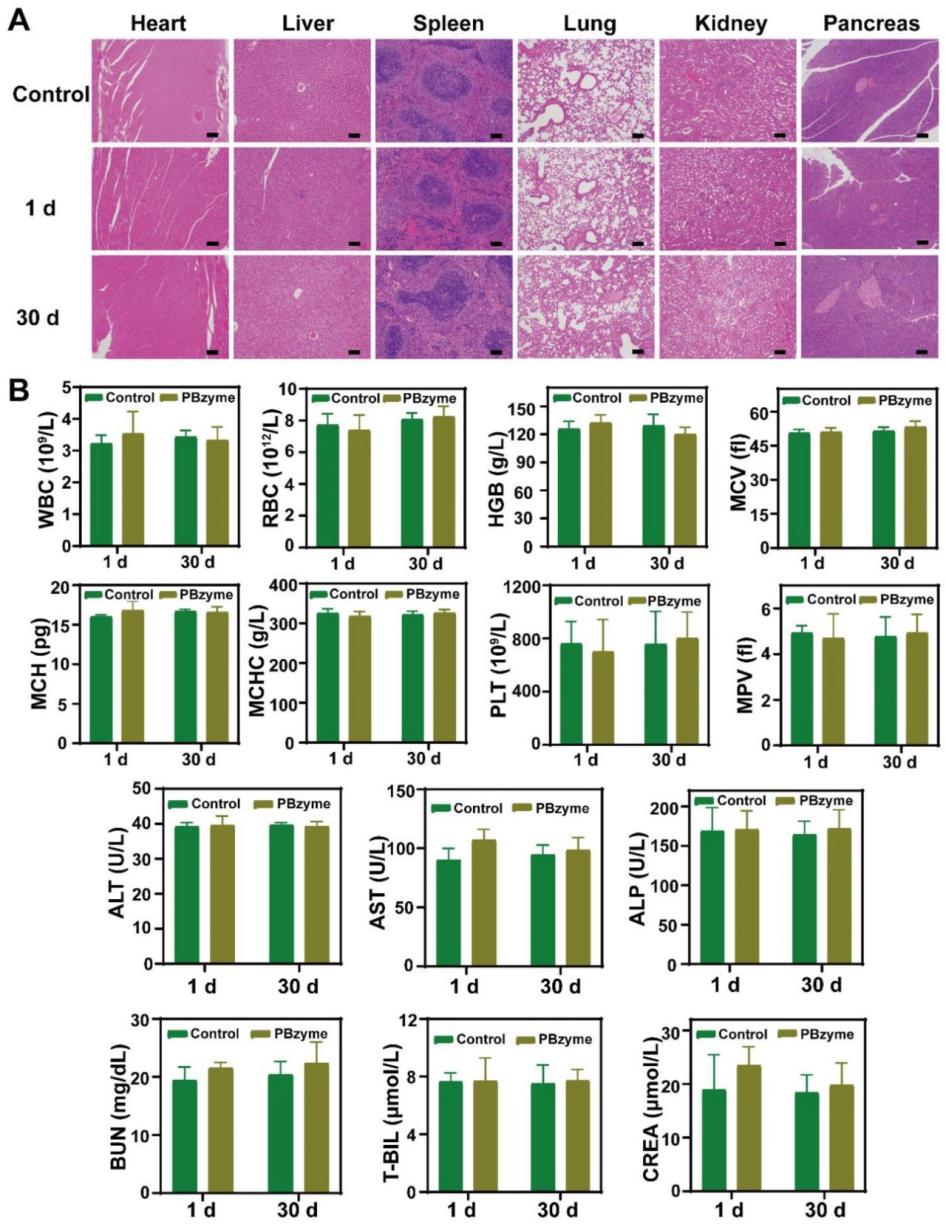
*In vivo* biosafety of PBzyme after intraperitoneal injection. (A) Histological sections of the heart, liver, spleen, lungs, kidneys, and pancreas in the mice after PBzyme intraperitoneal administration at different time points. Scale bars: 100 μm. (B) Hematological assays of mice after intraperitoneal injection of PBzyme at different time points.

Biosafety evaluation of nanodrugs is important for their clinical transformation. H&E staining showed no obvious change in mice treated with PBzyme (**Figure 7A**). In addition, blood indices including blood routine indices (WBC, RBC, HGB, MCV, MCH, etc) and blood biochemical indices (ALT, AST, ALP, BUN, etc) showed no significant difference among the control and PBzyme groups (**Figure 7B**).

## Conclusions

In summary, the detailed therapeutic mechanism of PBzyme with anti-oxidation and anti-inflammatory bioactivities in the preventive treatment of AP was revealed. PBzyme drives intrinsic anti-oxidative and anti-inflammatory bioactivities to achieve good preventive therapeutic efficacy for AP by inhibiting TLRs/NF-κB signaling pathways related to inflammation and oxidative stress, and by scavenging ROS. The effect and possible mechanism of PBzyme in alleviating the progression of AP are consistent with some reported drug mechanism in the treatment of AP, and also play a role by acting on the TLRs/NF-κB pathway. Compared with the currently reported drugs for the treatment of AP, PBzyme has the advantage of convenient preparation and *in vitro* preservation. The monodisperse PBzyme can be produced at the gram level by optimizing the preparation process. The single component, gram-level mass production, stable intrinsic bioactivities, biosafety, and good preventive therapeutic efficacy suggest that PBzyme is an ideal candidate in the preventive treatment of AP. Additional studies on the preventive therapeutic efficacy, long-term biosafety, and mechanism of PBzyme should be performed in an AP non-human primate model. Our results provide a foundation for the clinical application of PBzyme for AP as well as other ROS/inflammation-related diseases.

## Supplementary Material

Supplementary figures and table.

## Abbreviations

AP: acute pancreatitis
IL-1β: interleukin-1β
TNF-α: tumor necrosis factor-α
H_2_O_2_: hydrogen peroxide
ROS: reactive oxygen species
SOD: superoxide dismutase
PB: prussian blue
PBzyme: Prussian blue nanozyme
PCR: polymerase chain reaction
TEM: transmission electron microscope
SEM: scanning transmission electron microscope
XRD: X-ray diffraction
XPS: X-ray photoelectron spectroscopy
FTIR: fourier transform infrared spectroscopy
DLS: dynamic light scattering
ESR: electron spin resonance
•OH: hydroxylradical
•OOH: superoxide anion
XOD: xanthine oxidase
H_2_O_2_: hydrogen peroxide
ELISA: enzyme-linked immunosorbent assay
ALT: glutamic pyruvic transaminase
AST: glutamic oxaloacetic transaminase
CREA: creatinine
CTPO: 3-carbamoyl-2,2,5,5-tetramethyl-3-pyrroline-1-yloxyl
AMS: amylase
LPS: lipase
MDA: malondialdehyde
GSH: glutathione peroxidase
MCP-1: monocyte chemotactic protein 1
H&E: hematoxyli n and eosin staining
KEGG: Kyoto Encyclopedia of Genes and Genomes
TLR: Toll-like receptor
