# Supplementary material for: Prussian blue nanozyme-mediated nanoscavenger ameliorates acute pancreatitis via inhibiting TLRs/NF-κB signaling pathway
Source: Theranostics. 2021 Jan 1;11(7):3213–28. doi: 10.7150/thno.52010 (PMC7847676; doi:10.7150/thno.52010)
Supplement: Supplementary file 1 — Supplementary figures and table. [file thnov11p3213s1.pdf]

Supporting Information

Prussian Blue Nanozyme-mediated nanoscavenger treats acute pancreatitis via inhibiting TLRs/NF-κB signaling pathway

Xue Xie[1,4], Jiulong Zhao[2], Wei Gao[1], Jie Chen[3✉], Bing Hu[3], Xiaojun Cai[1✉], Yuanyi Zheng[1,3✉]

1. Department of Ultrasound in Medicine, Shanghai Jiao Tong University Affiliated Sixth People's Hospital, Shanghai, 200233, P. R. China
2. Department of Gastroenterology, Changhai Hospital, Second Military Medical University, Shanghai 200433, P. R. China.
3. Shanghai Institute of Ultrasound in Medicine, Shanghai Jiao Tong University Affiliated Sixth People's Hospital, Shanghai, 200233, P. R. China.
4. Chongqing Key Laboratory of Ultrasound Molecular Imaging, Ultrasound Department of the Second Affiliated Hospital of Chongqing Medical University. Chongqing 400010, P. R. China.

✉ Corresponding author: Yuanyi Zheng, PhD, Prof. E-mail: zhengyuanyi@sjtu.edu.cn; Jie Chen, PhD, E-mail: 808393@163.com; Xiaojun Cai, PhD, E-mail: c1x2j34@163.com.

* Xue Xie, Jiulong Zhao and Wei Gao contributed equally to this work.

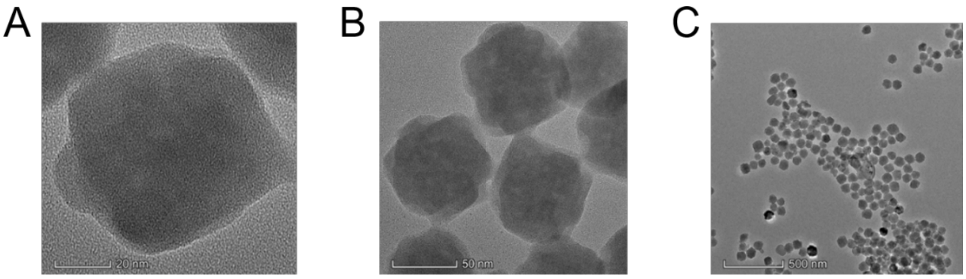

Figure S1. Transmission electron microscope image of PBzyme in various magnification.

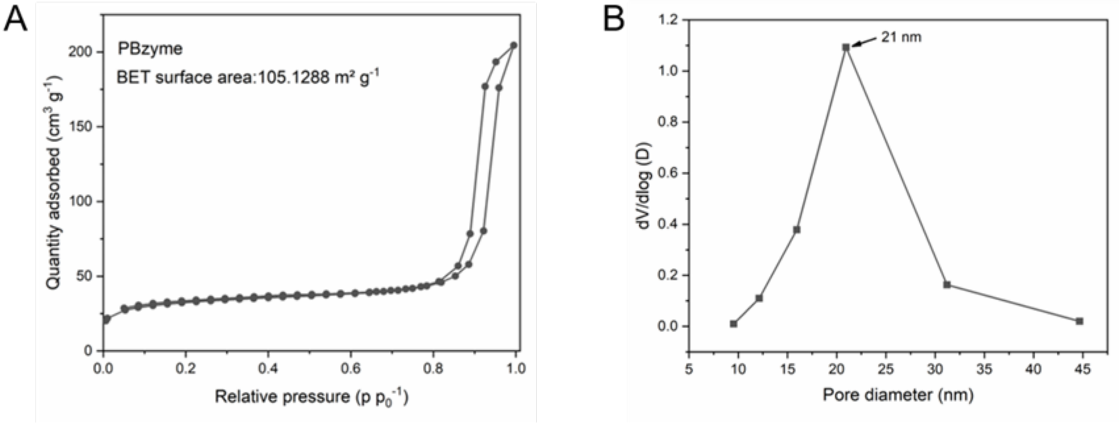

Figure S2. (A) N2 adsorption-desorption isotherms of PBzyme. (B) The pore size distribution of PBzyme.

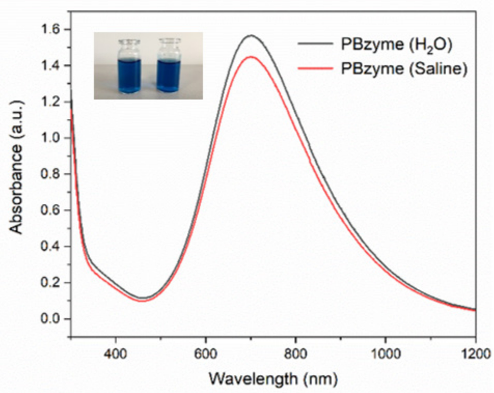

Figure S3. The UV-vis-NIR absorbance of PBzyme dispersed in water and normal saline. (Illustration: digital photographs of PBzyme in water and normal saline).

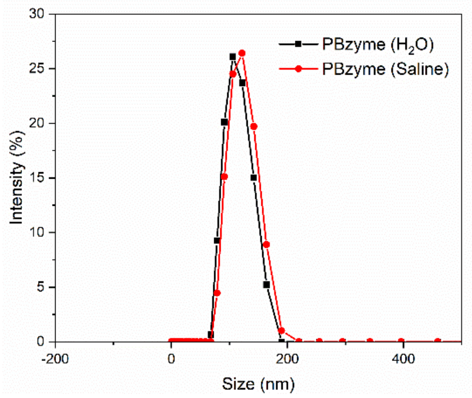

Figure S4. The hydrodynamic diameters of PBzyme in water and normal saline.

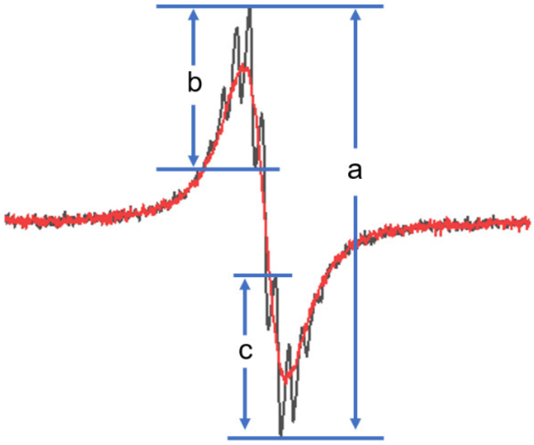

Figure S5. O_2 generation from PBzyme-catalyzed H_2O_2 degradation by ESR oximetry. The nitrogen nucleus shows ESR triplet spectrum in CTPO. The ESR spectrum of CTPO shows proton hyperfine structure in nitrogen saturated solution (black line), with the increase of oxygen concentration, the collision frequency between oxygen and nitroxide radical increases, the ESR triplet spectrum broadens, and the resolution of proton hyperfine structure decreases(red line). The change of oxygen concentration in the solution can be detected by calculating K value ($k = (b+c)/2a$) [1]. The smaller the k value is, the higher the oxygen concentration is.

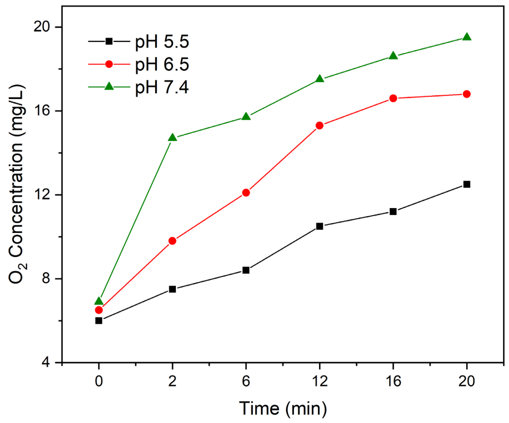

Figure S6. Oxygen generation catalyzed by PBzyme increased gradually with time at pH5.5, pH6.5 and pH7.4.

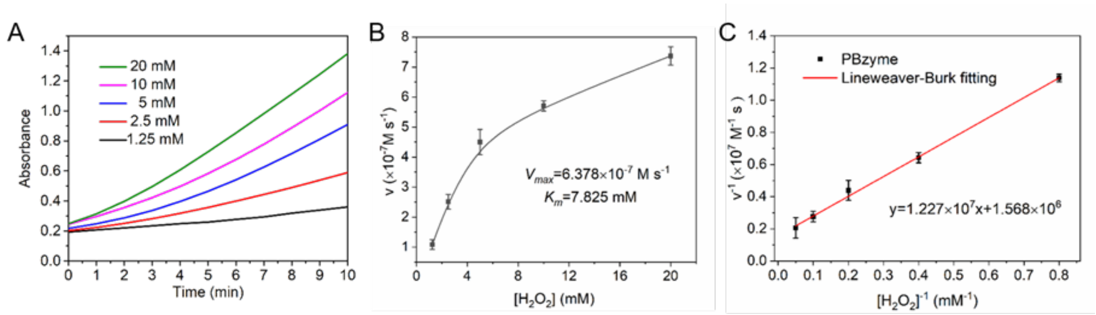

Figure S7. In vitro characterizations of the catalytic performance of PBzyme with H_2O_2. (A) The absorbance changes of PBzyme with different concentration of H_2O_2 at different time points. (B) Michaelis-Menten fitting curve of PBzyme with H_2O_2 as substrate. (C) Lineweaver-Burk plotting for PBzyme with H_2O_2 as substrate.

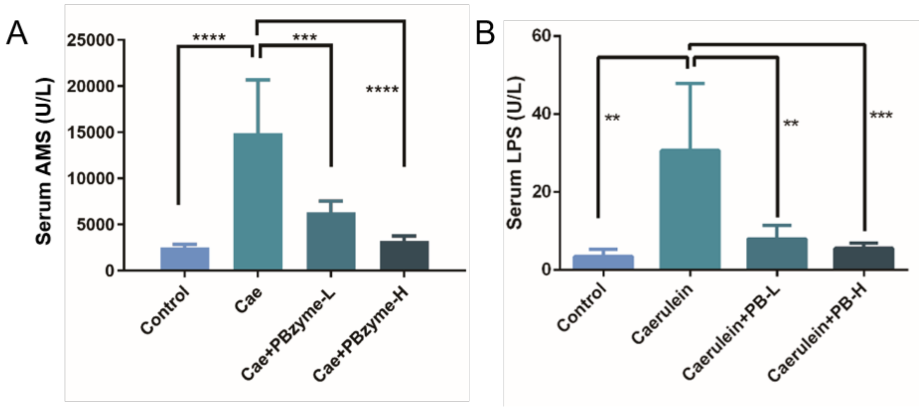

Figure S8. The levels of (A) serum AMS; and (B) serum LPS changed in various groups. (*, $p < 0.05$; **, $p < 0.01$; ***, $p < 0.001$, ****, $p < 0.0001$).

Schmidt pancreatic histopathological scoring standard

Pathological changes	Score			
	0	1	2	3
Interstitial edema	None	Interlobular	Intralobular	Acinus manifest islands
Inflammatory infiltration	None	<20	20-50	>50
Parenchymal necrosis	None	<5%	5-20%	>20%
Parenchymal hemorrhage	None	1-2	3-5	>5

Figure S9. Schmidt pancreatic histopathological score standard to evaluate the pancreatic histopathological score index of each group.

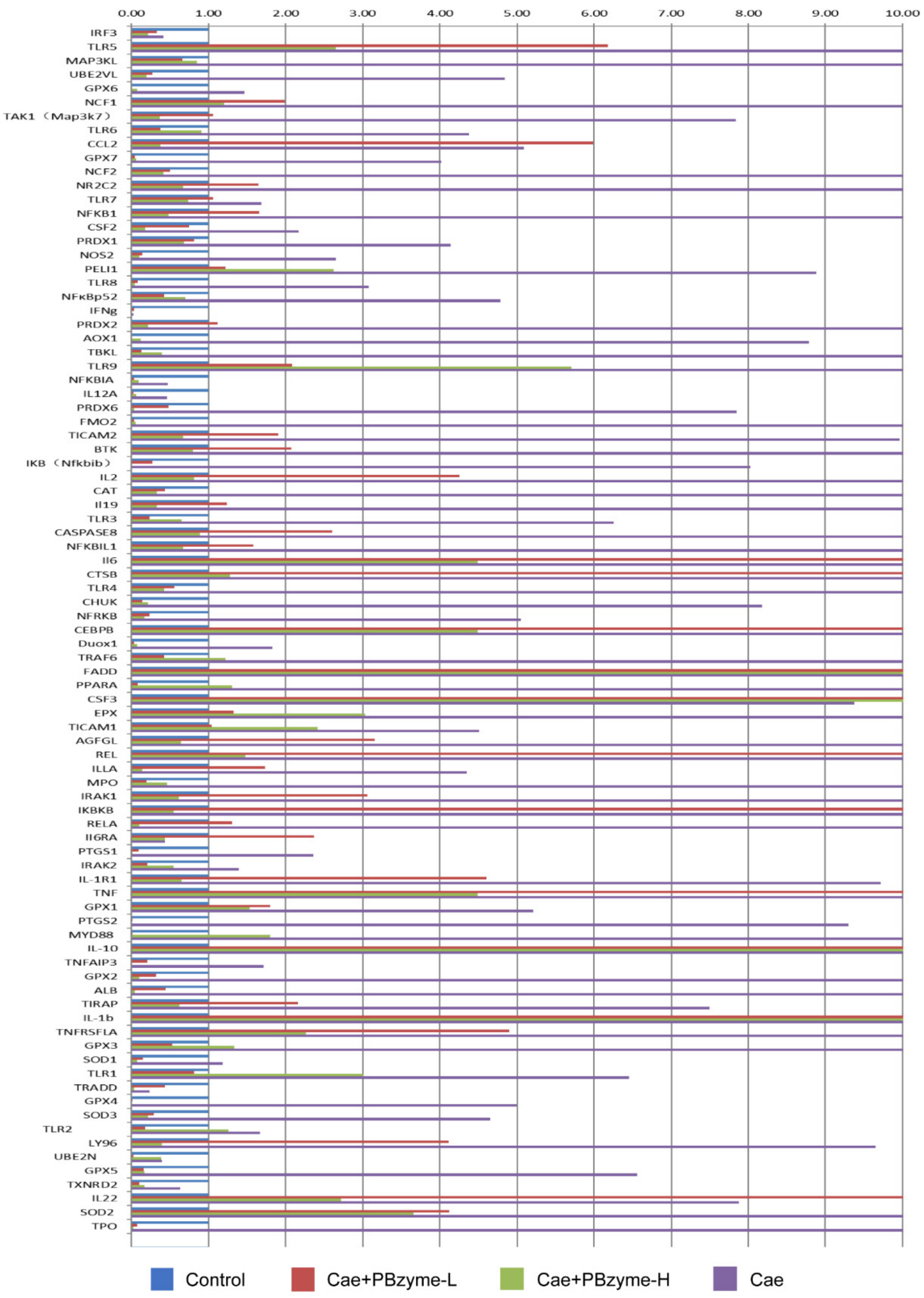

Figure S10. Cytokine profiles from a multiplexed cytokine assay of mice in the various groups (Control, Caerulein, Caerulein+PBzyme-L and Caerulein+PBzyme-H).

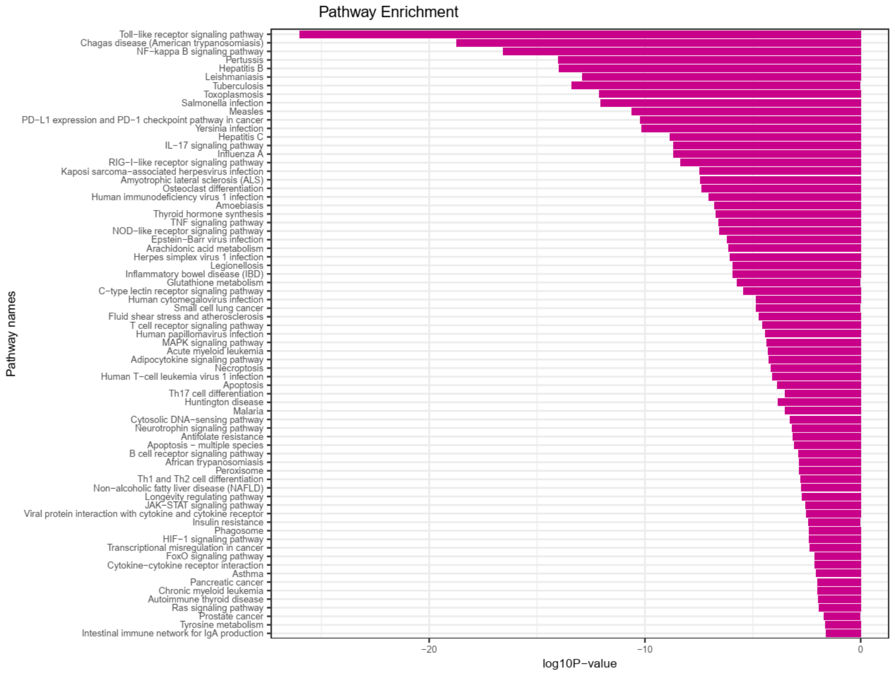

Figure S11. KEGG pathway analysis of the effect of PBzyme on caerulein-induced acute pancreatitis in mice. The name and reference sequence (Ref Seq) of target genes in inflammation-related and oxidation-related signaling pathways from the National Biotechnology Information Center (NCBI).

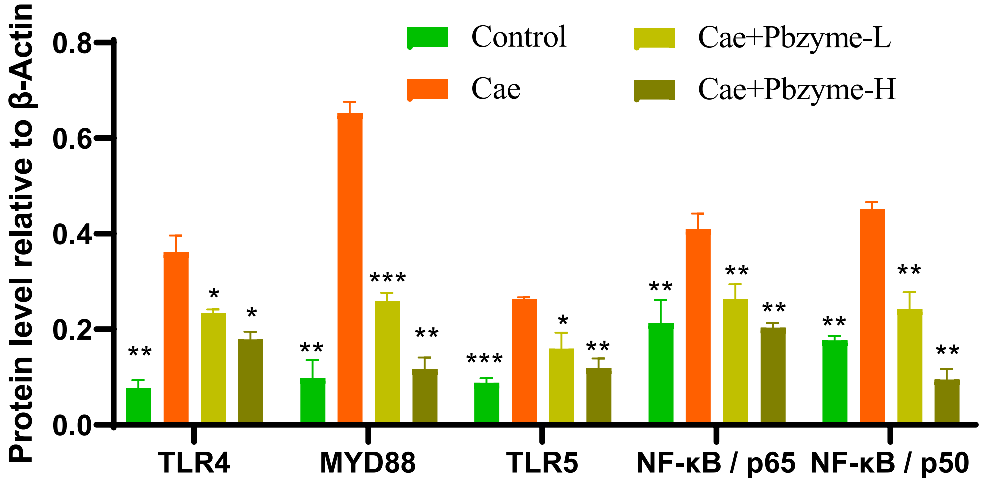

Figure S12. The quantification analysis of the protein levels of TLR4, MYD88, TLR5, NF-κB/p65 and NF-κB/p50 related to Toll-like receptor (TLR) and NF-κB signaling pathway in various groups. (n = 3) (*, $p < 0.05$; **, $p < 0.01$; ***, $p < 0.001$).

Number	Gene Name	RefSeq	Number	Gene Name	RefSeq
1	IRF3	NM_016849.4	45	DUOX1	NM_001099297.1
2	TLR5	NM_016928.2	46	TRAF6	NM_009424.3
3	MAP3KL	NM_011945.2	47	FADD	NM_010175.5
4	UBE2VL	NM_001311131.1	48	PPARA	NM_001113418.1
5	GPX6	NM_145451.3	49	CSF3	NM_009971.1
6	NCF1	NM_001286037.1	50	EPX	NM_007946.2
7	TAK1（Map3k7）	NM_009316.1	51	TICAM1	NM_174989.4
8	TLR6	NM_011604.3	52	AGFGL	NM_001310713.1
9	CCL2	NM_011333.3	53	REL	NM_009044.2
10	GPX7	NM_024198.3	54	ILLA	NM_010554.4
11	NCF2	NM_010877.5	55	MPO	NM_010824.2
12	NR2C2	NM_001347342.1	56	IRAK1	NM_001177973.1
13	TLR7	NM_133211.3	57	IKBKB	NM_001159774.1
14	NFκB1	NM_008689.2	58	RELA	NM_009045.4
15	CSF2	NM_009969.4	59	IL6RA	NM_001310676.1
16	PRDX1	NM_011034.4	60	PTGS1	NM_008969.4
17	NOS2	NM_010927.3	61	IRAK2	NM_001113553.1
18	PELI1	NM_023324.2	62	IL1R1	NM_001123382.1
19	TLR8	NM_133212.2	63	TNF	NM_013693.2
20	NFκBp52	NM_001177369.1	64	GPX1	NM_008160.6
21	IFNG	NM_008337.3	65	PTGS2	NM_011198.3
22	PRDX2	NM_011563.5	66	MYD88	NM_010851.2
23	AOX1	NM_009676.2	67	IL10	NM_010548.2
24	TBKL	NM_019786.4	68	TNFAIP3	NM_001166402.1
25	TLR9	NM_031178.2	69	GPX2	NM_030677.2
26	NFκBIA	NM_001105720.2	70	ALB	NM_009654.4
27	IL12A	NM_001159424.1	71	TIRAP	NM_001177845.1
28	PRDX6	NM_007453.3	72	IL1B	NM_008361.3
29	FMO2	NM_018881.3	73	TNFRSFLA	NM_011609.4
30	TICAM2	NM_173394.3	74	GPX3	NM_008161.3
31	BTK	NM_013482.2	75	SOD1	NM_011434.1
32	IKB（Nfkbib）	NM_001306222.1	76	TLR1	NM_030682.1
33	IL2	NM_008366.3	77	TRADD	NM_001033161.2
34	CAT	NM_012520.1	78	GPX4	NM_001037741.3
35	Il19	NM_001009940.1	79	SOD3	NM_011435.3
36	TLR3	NM_126166.4	80	TLR2	NM_011905.3
37	CASPASE 8	NM_009812	81	LY96	NM_001159711.1
38	NFκBIL1	NM_010909.4	82	UBE2N	NM_080560.3
39	Il6	NM_031168.1	83	GPX5	NM_010343.2
40	CTSB	NM_007798.3	84	TXNRD2	NM_013711.3
41	TLR4	NM_021297.2	85	IL22	NM_016971.2
42	CHUK	NM_001162410.1	86	SOD2	NM_013671.3
43	NFRκB	NM_172766.3	87	TPO	NM_009417.2
44	CEBPB	NM_024125.4	88	β-actin	NM_007393.5

Table S1. The Gene Names and Reference Sequence (Ref Seq) mostly related to inflammation and oxidation signaling pathways from National Center for Biotechnology Information (NCBI).

REFERENCES

[1] Zeng F, Wu Y, Li X, et al. Custom-Made Ceria Nanoparticles Show a Neuroprotective Effect by Modulating Phenotypic Polarization of the Microglia. Angew Chem Int Ed Engl. 2018;57:5808-12.
